# Cognitive Advantages of Multilingual Learning on Metalinguistic Awareness, Working Memory and L1 Lexicon Size: Reconceptualization of Linguistic Giftedness from a DMM Perspective

**DOI:** 10.5334/joc.201

**Published:** 2022-01-07

**Authors:** Ferhat Dolas, Ulrike Jessner, Gülay Cedden

**Affiliations:** 1 Department of English, University of Innsbruck, Innsbruck, Austria; 2 Department of Applied Linguistics, University of Pannonia, Hungary, AT; 3 Department of Foreign Language Teaching, Middle East Technical University, Ankara, Turkey

**Keywords:** multilingualism, metalinguistic awareness, cognition, working memory, giftedness, linguistic giftedness

## Abstract

The relation between multilingual learning and cognition through (linguistic) giftedness has not been studied yet in third language acquisition, multilingualism or cognition studies. Even though ‘giftedness’ appears to be enigmatic and advantageous in a number of areas, in the field of language learning it is not clear whether multilingual learning or giftedness fulfils the triggering role in a number of cognitive skills. For that purpose, the present study observed the possible cognitive advantages of multilingual learning on metalinguistic awareness (Jessner 2006), working memory (Baddeley & Hitch 1974; Robinson 2002; 2012) and first language lexicon size of a number of children from regular and gifted education programmes in a Dynamic Model of Multilingualism perspective (Herdina & Jessner 2002). The study was analyzed with the multiple linear regression model based on the scores gathered from the data of working memory and vocabulary sub-tests of the Turkish adaptation version (Savaşır & Şahin 1995) of the Wechsler Intelligence Scale for Children—Revised, and metalinguistic awareness test (Pinto et al. 1999) of a number of mono-, bi- and multilingual participants from various schools. The results not only provided positive correlations between multilingual learning and metalinguistic awareness, working memory and first language lexicon size but also contributed to the identification and reconceptualization of linguistic giftedness.

## Introduction

During the last decades the bilingual and multilingual advantages over monolinguals have forced psycholinguistic studies to focus on the relation between language(s) and cognition and mental organisation of two or more linguistic systems. A number of scholars such as Bialystok (2012; 2014), Herdina and Jessner (2002), Jessner (2018), Biedron (2015) have already stated the positive effects of bilingualism and multilingualism on development of language and cognition. Jessner (2006) also highlights that cognitive advantages of bi- and multilinguals over monolinguals are often related to an increased level of metalinguistic awareness (MeLA). Lexicon size is regarded as a major factor in language acquisition and strongly related to metalinguistic skills as well (see Altman et al. 2018). Related to the present study, Adesope et al. (2010) point that further work investigating the cognitive correlates of bilingualism within educational contexts is required to clarify the advantages of bi- and multilingualism in practice.

In the frame of linguistic giftedness (LG) of the present study, Biedron and Pawlak (2016) underline that in the field of second and third language acquisition (SLA & TLA) hardly any research stating gifted and exceptionally talented language learners has been accomplished and as a result little is known about this rare population. Scholars such as Ameringer et al. (2018) mention that foreign language aptitude is a term that subsumes a number of concepts and is often used interchangeably with other terms, such as talent, giftedness, language learning ability or even sometimes with language learning expertise. However, in linguistics and psycholinguistics there has not been a model specifically developed to consider the dimensions of LG. This situation causes vagueness in identifying the boundary between IQ giftedness and LG. Additionally, even though ‘giftedness’ appears to be advantageous in a number of areas, in the field of language learning it is not clear whether multilingual learning or giftedness fulfils the triggering role in cognition. Consequently, the present study specifically focuses on the relation between multilingual learning and cognition through linguistic giftedness at educational contexts.

## What is Giftedness?

The key criterion in determining gifted children is generally intelligence score-total or general Intellectual/Intelligence Quotient (IQ) score. Accordingly, a child obtaining a total IQ higher than or equal to 130 is identified as gifted (Sattler 2002; Pfeiffer 2012). However, a number of scholars agree that IQ cannot be used as a single variable in the conceptualization of high abilities (Calero & García-Martín 2011; Pfeiffer 2015). Fernández et al. (2017) state that IQ remains an important factor to be assessed and, when used in conjunction with other variables it can provide essential information concerning the identification of students with exceptional abilities (Sternberg 2010; Renzulli & Gaesser 2015; cited in Fernandez et al. 2017). The term *giftedness* can also be synonymously used for *aptitude* and refers to an undeveloped, biologically inherited predisposition for acquiring a certain skill (see Biedron & Pavlak 2016). High aptitudes become well-trained skills (expertise) that are systematically developed (Gagné 2005; Seither-Preisler, Parncutt & Schneider 2014). In years, definitions that consider giftedness as potentially trainable (Sternberg 2002; Dweck 2006; Mercer 2012) have made a distinction between what a child is capable of achieving and what the child will achieve. In this frame, it can be stated that the *potential to be developed* indicates the dynamic and developmental structure of the potential itself which is also highlighted in the Dynamic Model of Multilingualism (DMM) by Herdina and Jessner (2002).

## Linguistic (Verbal) Giftedness

The most significant characteristics of exceptionally successful learners is unusual verbal memory (Biedron & Pawlak 2016). Indeed, outstanding memory for verbal material has been the most striking characteristics of all the described cases of talented individuals (cited in Biedron & Pawlak 2016; see Novoa et al. 1988; Schneiderman & Desmarais 1988; Ioup et al. 1994; Erard 2012). Bailey (1996: 97) defines verbally gifted children as those who demonstrate at an early age, complex behaviours in listening, speaking, reading and writing. These children have a “true agility” in manipulating linguistic symbols as well as the codes necessary for turning thought into expression or in the case of reading, expression into thought (Bailey 1996: 101). According to Biedron (2016), all linguistically gifted individuals seem to share common cognitive characteristics, such as excellent memory, especially working memory (WM) enables them to acquire verbal material faster and easier than less gifted individuals (McCabe et al. 2010). It is often stated that WM plays a role in determining the outcome of foreign language learning (Dörnyei 2005; Ellis 2001; Miyake & Friedman 1998; Robinson 2009; Sawyer & Ranta 2001). In this frame, it should be underlined that an outstanding memory for verbal material is the most striking characteristic of talented individuals (see Novoa et al. 1988; Schneiderman & Desmarais 1988; Ioup et al. 1994; Erard 2012).

In a study on the effectiveness of an integrated language arts curriculum by vantassel-Baska et al. (1996), it was found that verbally gifted children were able to increase their linguistic competence. Scholars such as Ameringer, Green, Leisser and Turker (2018) state that foreign language aptitude is a term that subsumes a number of concepts and is often used interchangeably with other terms, such as talent, giftedness, language learning ability or even sometimes with language learning expertise. The same authors also add that although it is often still difficult to know where to draw the line and differentiate the variety of terms, researchers have at least suggested a differentiation between talent and aptitude according to which aptitude designates the innate property that develops into a certain skill which is then termed *talent* (see Gagné 1995; 2005; Stern & Neubauer 2013).

## The Relation between Cognition and Language

The relation between language and cognition depending on a dispute whether these two are independent mental capacities or language derives from cognitive skills has been questioned for decades. One crucial point is whether cognitive skills are affected from language(s) related abilities. In contrast to formal linguistic studies, which claim that the language faculty is a module independent from other cognitive modules and ruled by linguistic mechanisms, psycholinguistic scholars essentially claim that the processes of language acquisition are the same as those used in the acquisition of any other cognitive skills such as mathematical abilities, where cognitive factors such as memory, attention/perception, intelligence etc. are at work (see Mayo 2011).

From a DMM perspective, the cognitive advantages of multilinguals are often related to an increased level of MeLA (Herdina & Jessner 2002; Jessner 2006; 2008; 2018). Herdina & Jessner (2002) also discuss the features of multilingual development starting with the implicit linear model of language acquisition which considers language learning first, second or third as gradual sequence of language improvement leading to an acceptable degree of mastery of a language system. Gombert (1992) viewed metalinguistic activities as a subfield of metacognition and argues that metalinguistic reflection may result in cognitive products or symbolic objects which are easily perceived and frequently manipulated by the child and which are important for the general development of thought and more specifically for metacognitive development. Furthermore, an increased level of MeLA seems to be characteristic of bi- and multilingual development (Bialystok 2001). Biedron (2015) states that the process of constant switching from one language to another and constant operating in two linguistic code systems facilitates a dual linguistic perspective. That bilinguals are more metalinguistically aware which makes them more cognitively advantageous and flexible (see Bialystok 1986; 1988; 2001; 2009; Bialystok & Majumder 1998; Costa et al. 2008; Davidson et al. 2010; Green 1998; Jessner 1999; 2006; 2018; Biedron 2015) alters perceptions on multilingual minds so that they are supposed to be more adaptable compared to mono- and bilinguals. In a similar vein, Singleton and Aronin (2007: 83) hypothesise that multilinguals have a more extensive range of affordances available. Thus, knowledge of more than one or two languages can support develop specific types of competence. That is, multilingualism generally gives the impression to help people realise and expand their creative potential in ability to communicate in various occasions by using a number of interrelated and complex linguistic, cognitive components. Kharkhurin (2009; 2012) argues that there is a link between bilingualism/multilingualism and creativity as well.

Moreover, WM, which has been assumed to play a central role in a wide range of cognitive activities (see Kane & Engle 2002; Alloway et al. 2004), is a term adapted from cognitive psychology which generally refers to our ability to maintain and operate on a limited amount of information when doing some mentally demanding tasks (Baddeley 2015). Żelechowska et al. (2017) state that WM is investigated as a possible determinant of complex cognitive processes such as thinking and problem solving or complex cognitive skills such as intelligence or language proficiency due to its functions. Robinson (2003) mentions that WM is an important contributor to second language learning ability. WM in general has been argued to be strongly implicated in aptitude for L2 processing and language learning (cited in Robinson 2012; Miyake & Friedman 1998; Ellis 2001; Robinson 2002a; Robinson 2000b; Robinson 2005; Williams & Lovatt 2003). Working memory (WM) is also regarded as characteristics of intellectual giftedness (Hoard et al. 2008; Kornmann, Zettler, Kammerer, Gerjets & Trautwein 2015; Vock & Holling 2008). On the other hand, according to Alloway and Elsworth (2012) the correlation between IQ and working memory capacity tends to decrease as intelligence increases.

## Aims of the Study

The present study aimed to observe the possible cognitive advantages of multilingual learning on metalinguistic awareness, working memory and first language lexicon size of a number of children from regular and gifted education programmes in a Dynamic Model of Multilingualism perspective (Herdina & Jessner 2002). As already stated above, from a DMM perspective, the cognitive advantages of multilinguals are often related to an increased level of MeLA. In this frame, possible predictors of metalinguistic awareness were observed initially in order to clarify the potential effects of the independent variables of WM, lexicon size and number of the languages learnt. For the second step, the possible correlations between variables were observed. In third and final step, it was aimed to find out whether multilingual learning or IQ giftedness was a better predictor of high MeLA, WM and L1 lexicon size test scores.

## Method

The study was conducted with 117 participants of two groups aged between 11 and 14. The groups were structured according to the participating schools’ language(s) teaching curricula. The first group (n = 81) was from regular schooling programme and the second group was from a gifted programme (n = 36). In both groups the participants were grouped as monolinguals (n = 25, n = 9), bilinguals (n = 36, n = 14) and multilingual learners (n = 20, n = 13). The monolinguals and bilinguals were from state and private schools where the language of instruction is Turkish. The bilinguals had one year English immersion programme. The multilingual learners in the first group were from a multilingual (IB-International Baccalaureate) school where English is the language of bilingual education and German is a compulsory third language. Spanish, Latin and French are optional. The multilinguals of the 2.group were from private schools where the language of instruction is Turkish, additionally they had one year English immersion programme and learnt basic German as an optional third language. The descriptive statistics of participants are provided in ***Tables 1*** and ***2***.

**Table 1:** Number of the participants.


GENDER LANGUAGE	FEMALE	MALE	TOTAL

Monolingual	13	21	34

Bilingual	36	14	50

Multilingual	24	9	33

**Total**	**73**	**44**	**117**


**Table 2:** Descriptive Statistics.


	N	MINIMUM	MAXIMUM	MEAN	STD. DEVIATION

AGE	117	11	14	12,44	1,132

GENDER	117	*1,00	**2,00	1,3761	,48648

Valid N (listwise)	117				


*Female **Male.

## Procedure

WM and L1 lexicon size were tested through Digit Span, Picture Span and Arithmetic sub-tests of WM and Vocabulary sub-test of the Wechsler Intelligence Scale for Children—Revised (Wisc-R) which was adapted into Turkish by Savaşır and şahin (1995). In general, Wisc-R (Wechsler 1974), which is a revised and updated version of the WISC, attempts to measure intelligence by 12 subtests. Its scoring procedure results in a scaled score for each of these subtests (Franzen 2000). The scaled scores are combined to produce scores for Verbal IQ (VIQ), Performance IQ (PIQ) and Full Scale IQ (FSIQ) (Franzen 2000). The participants were examined individually and the scaled scores were combined to produce working memory index (WMI) scores. According to Wisc-R WM test application (Wechsler 1974; Savaşır & şahin 1995), for the digit span test there is not an attended time limit due to the fact that it is a quick response repetition test. For the picture span for the sets between 3 and 8 the time limit is 45 seconds per set and for the sets between 10 and 12 the time limit is 60 seconds per set. For the arithmetic test the questions between 8 and 13 have 30, 14 and 15 have 45 and 16 and 18 have 75 seconds per question. The productive lexicon size test was administered separately as a one page paper-pen form. Although no time limit is attended for the vocabulary test, it observed that responses were generally completed in 5 to 7 minutes.

In order to measure MeLA, Metalinguistic Awareness Test (MAT-2) by Pinto et al. (1999), which is an instrument for the measurement of metalinguistic ability and awareness, was translated into Turkish and adapted it into Turkish context. The test originally has six sections; Comprehension, Synonymy, Acceptability, Ambiguity, Grammatical Function and Phonemic Segmentation. The tests were given a maximum of 50 minutes, while the synonymous and grammatical functions sections were limited to 30 minutes. Each section of L(inguistic) and the M(eta)L(inguistic) areas were coded differently as in the original. The L responses were quoted according to the right or wrong dichotomous procedure (1 or 0 point). The total score of each section was constructed by summing up scores of the individual items (226 for the adapted test). The ML responses were evaluated item by item in three levels. The total score of each section was constructed by adding the scores of the individual items. The qualitative characteristics underlying the mentioned ML levels valid for the first five sections and partially for the phonemic segmentation test were as follows: Level 0: Pre-analytic level: The subject could not analyze the sum of the semantic indexes and grammar in the presented items. Level 1: Relevant but insufficient analysis: The subject used a crude method of analysis, isolating, for example, at least one of the semantic-grammatical clues, or rewrapping the content of the item as a relevant paraphrase. The arguments given to the answers were not sufficient to resolve the ambiguity that the sentence contains. Level 2: Pertinent and exhaustive analysis: The subject used a systematic method of analysis, identifying all relevant semantic and grammatical indices in the item (MAT-2 by Pinto et al. 1999).

## Analyses

### 1. Study with Non-gifted Children

The multiple regression model was used to investigate which of independent variable(s) can predict a dependent variable in both study groups. The independent variables were defined as WM, lexicon size and the number of languages learnt (mono-, bi- or multilingual) and the dependent variable was MeLA. ***Tables 3, 4, 5*** and ***6*** provide detailed information about the regression model. Among the assumptions of multiple regressions for the current study; the Durbin-Watson value was found as 1.922 that indicated the residuals were uncorrelated. The dependent variable MeLA was normally distributed taking the non-significant p value (p = 204 > 0.05) of Shapiro-Wilk. In the model summary r = .732 and *r*^2^ = .536 indicated that 53.6 of the variance of the dependant variable was explained by the independent variables. In the correlation table the highest *r = .620* value was between multilingualism and MeLA, the lowest *r = –.461* value between monolinguals and MeLA. A positive correlation between MeLA and WM was (= .336), MeLA and vocabulary size was (= .562) and MeLA and multilingualism was (= .620). In the Anova table (p = .000) was observed as an indication of significance. The multicollinearity assumption was also checked by the Cook’s distance (= .179) value and the standard residual (–2.543 and +2390) and there was no multicollinearity. In the coefficient table, (***Table 6***) multilingualism p = .000 < 0.05, WM p = .033 < 0.05 and lexicon size p = .000 < 0.05 were observed as significant predictors. In the model, lexicon size (β = .390), multilingualism (β = .412) and WM (β = .183) were found as contributing predictors.

**Table 3:** Model Summary^b^ of multiple regression of non-gifted group.


MODEL	R	R SQUARE	ADJUSTED R SQUARE	STD. ERROR OF THE ESTIMATE	CHANGE STATISTICS					DURBIN-WATSON

					R SQUARE CHANGE	F CHANGE	DF1	DF2	SIG. F CHANGE	

1	**,732^a^**	**,536**	**,510**	**18,993**	**,536**	**20,546**	**4**	**71**	**,000**	**1,922**


a. Predictors: (Constant), MULTI, WM, VOC, MONO. b. Dependent Variable: MeLA.

**Table 4:** ANOVA^a^ table of the multiple regression of non-gifted group.


MODEL		SUM OF SQUARES	DF	MEAN SQUARE	F	SIG.

1	Regression	**29644,864**	**4**	**7411,216**	**20,546**	**,000^b^**

	Residual	**25611,175**	**71**	**360,721**		

	Total	**55256,039**	**75**			


a. Dependent Variable: MeLA b. Predictors: (Constant), MULTI, WM, VOC, MONO

**Table 5:** Correlations of the multiple regression model of non-gifted group.


		MELA	WM	VOC	MONO	BI	MULTI

Pearson Correlation	MeLA	**1,000**	**,336**	**,562**	**–,461**	**–,119**	**,620**

	WM	**,336**	**1,000**	**,067**	**–,348**	**,054**	**,307**

	VOC	**,562**	**,067**	**1,000**	**–,549**	**,171**	**,388**

	MONO	**–,461**	**–,348**	**–,549**	**1,000**	**–,579**	**–,406**

	BI	**–,119**	**,054**	**,171**	**–,579**	**1,000**	**–,510**

	MULTI	**,620**	**,307**	**,388**	**–,406**	**–,510**	**1,000**

Sig. (1-tailed)	MeLA	.	,002	,000	,000	,153	,000

	WM	,002	.	,284	,001	,323	,003

	VOC	,000	,284	.	,000	,070	,000

	MONO	,000	,001	,000	.	,000	,000

	BI	,153	,323	,070	,000	.	,000

	MULTI	,000	,003	,000	,000	,000	.


**Table 6:** Coefficients^a^ table of the multiple regression for non- gifted participants.


MODEL		UNSTANDARDIZED COEFFICIENTS		STANDARDIZED COEFFICIENTS	T	SIG.	CORRELATIONS		
	
		B	STD. ERROR	BETA			ZERO-ORDER	PARTIAL	PART

1	(Constant)	**47,290**	**10,215**		**4,629**	**,000**			

	WM	**,343**	**,158**	**,183**	**2,170**	**,033**	**,336**	**,248**	**,174**

	VOC	**,825**	**,184**	**,390**	**4,472**	**,000**	**,562**	**,466**	**,359**

	MULTI	**25,221**	**5,601**	**,412**	**4,503**	**,000**	**,620**	**,469**	**,361**


a. Dependent Variable: MeLA

**Figure 1:**
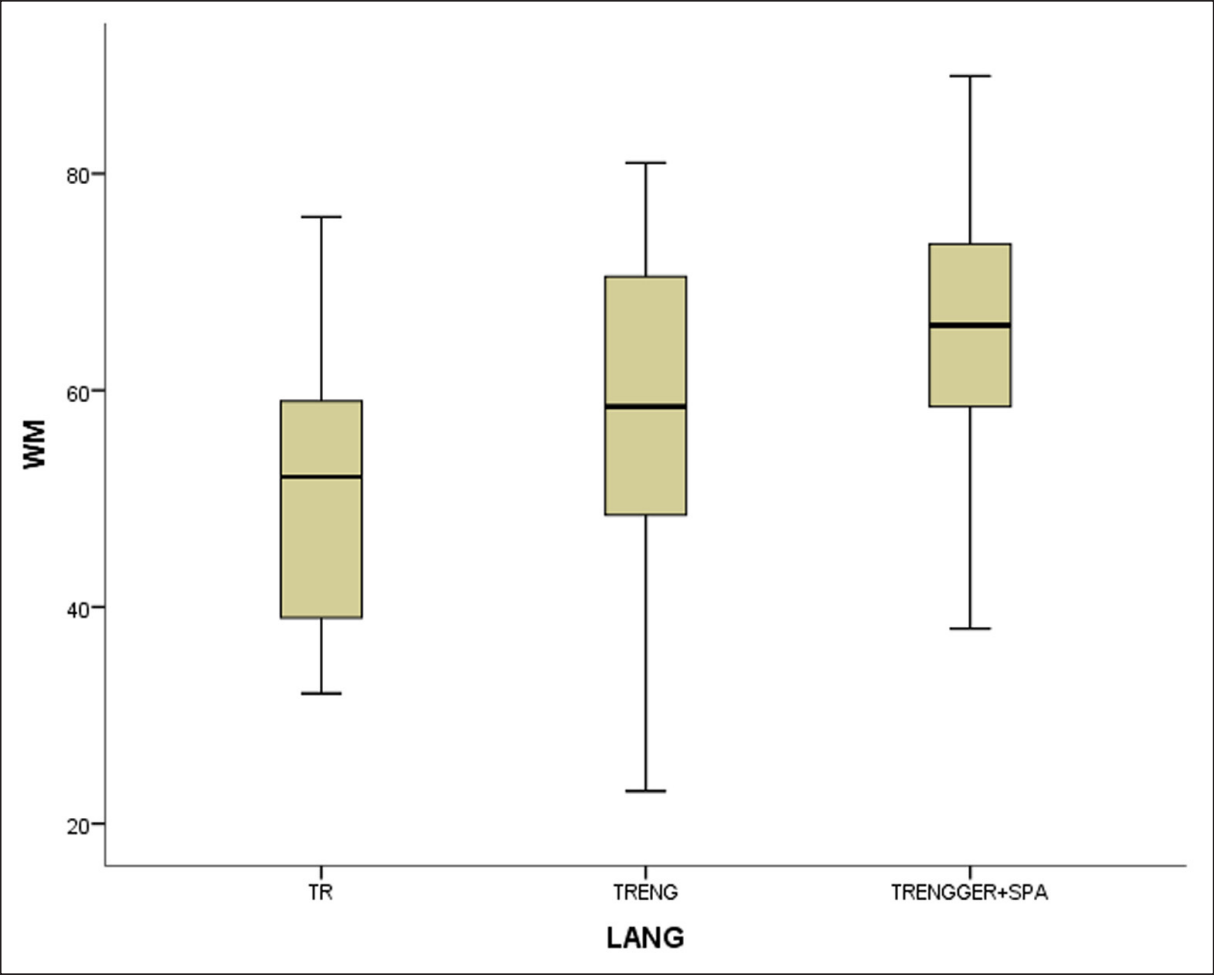
The boxplots of variables (WM for working memory, MeLA for metalinguistic awareness and VOC for vocabulary) in non-gifted group.

**Figure 2:**
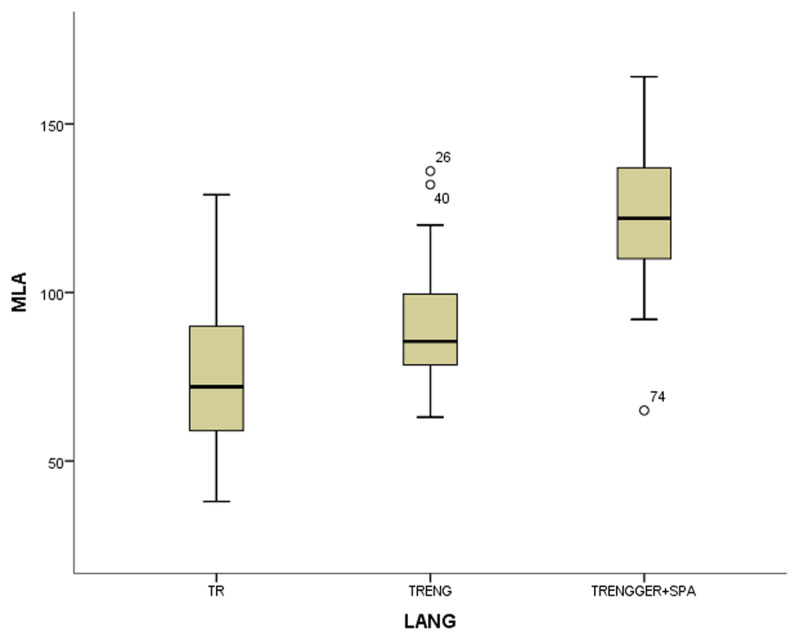
The boxplots of variables (WM for working memory, MeLA for metalinguistic awareness and VOC for vocabulary) in non-gifted group.

**Figure 3:**
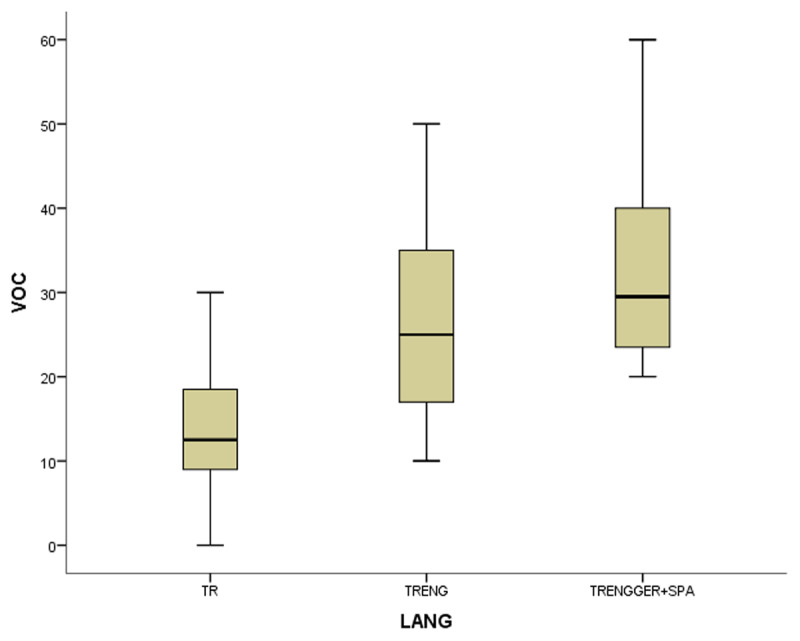
The boxplots of variables (WM for working memory, MeLA for metalinguistic awareness and VOC for vocabulary) in non-gifted group.

**Figure 4:**
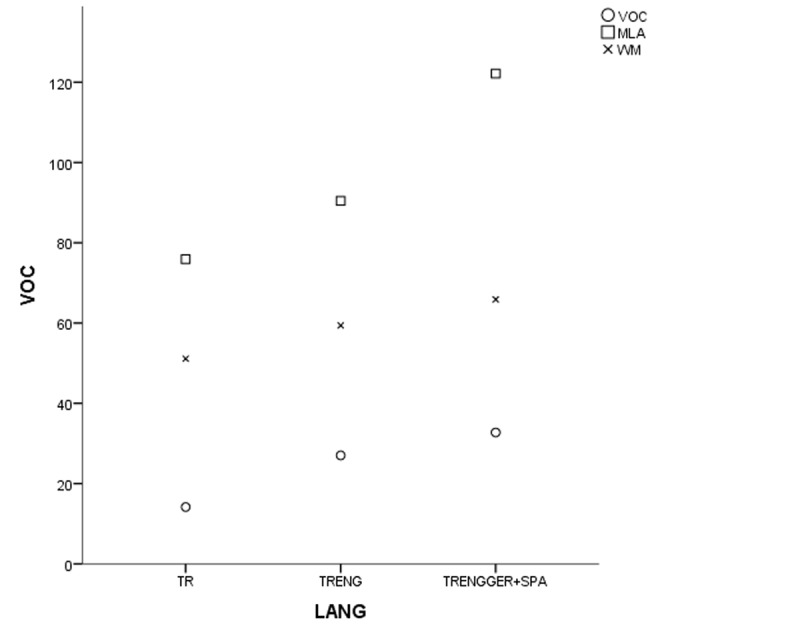
Grouped scatter-plot of the variables in non-gifted participants (TR for monolinguals, TRENG for bilinguals and TRENGGER+SPA for multilinguals).

In the coefficient table (***Table 6***) of the first model of non-gifted participants, multilingualism, working memory and vocabulary were observed as significant contributing predictors. Other explanatory variables of bi- and monolingualism did not provide substantial contributions to MeLA and were excluded in the current model. ***Figures 1, 2, 3*** and ***4***.

### 2. Study with Gifted Children

Among the assumptions in the second model; the Durbin-Watson value was found as 1.730 that indicated the residuals were uncorrelated. The dependent variable MeLA was normally distributed taking the non-significant p value (p = 749 > 0.05) of Shapiro-Wilk. In the model summary r = .667 and *r*^2^ = .445 indicated that 44,5 of the variance of the dependant variable was explained by the independent variables. In the correlation table the highest *r = .533* value between multilingualism and MeLA, the lowest *r = –.368* value between monolinguals and MeLA were observed. A positive correlation between MeLA and WM was (= .193), MeLA and vocabulary size was (= .511) and MeLA and multilingualism (= .553) was observed. In the Anova table (p = .000) was observed as an indication of significancy. The multicollinearity assumption was also checked by the Cook’s distance (= .172) value and the standard residual (–2.302 and +2.982) and no multicollinearity was observed. In the coefficient table, the variables of vocabulary size and multilingualism have p = .004 < 0.05 and p = .002 < 0.05 and can be concluded that they are significant predictors with VOC (β = .412), MULTI (β = .439). ***Tables 7, 8, 9*** and ***10*** provide a detailed information about the regression model.

**Table 7:** Model Summary^b^ of multiple regression of the gifted participants.


MODEL	R	R SQUARE	ADJUSTED R SQUARE	STD. ERROR OF THE ESTIMATE	CHANGE STATISTICS					DURBIN-WATSON

					R SQUARE CHANGE	F CHANGE	DF1	DF2	SIG. F CHANGE	

1	**,667^a^**	**,445**	**,411**	**11,706**	**,445**	**13,205**	**2**	**33**	**,000**	**1,730**


a. Predictors: (Constant), MULTI, VOC. b. Dependent Variable: MeLA.

**Table 8:** ANOVA^a^ table of the multiple regression for gifted participants.


MODEL		SUM OF SQUARES	DF	MEAN SQUARE	F	SIG.

1	Regression	**3827,143**	**3**	**1275,714**	**9,462**	**,000^b^**

	Residual	**4314,496**	**32**	**134,828**		

	Total	**8141,639**	**35**			


a. Dependent Variable: MeLA. b. Predictors: (Constant), MULTI, WM, VOC.

**Table 9:** Correlations of the multiple regression model of gifted participants.


		MELA	WM	VOC	MONO	BI	MULTI

Pearson Correlation	MeLA	**1,000**	**,193**	**,511**	**–,368**	**–,198**	**,533**

	WM	**,193**	**1,000**	**–,108**	**–,059**	**–,133**	**,188**

	VOC	**,511**	**–,108**	**1,000**	**–,239**	**–,011**	**,227**

	MONO	**–,368**	**–,059**	**–,239**	**1,000**	**–,461**	**–,434**

	BI	**–,198**	**–,133**	**–,011**	**–,461**	**1,000**	**–,600**

	MULTI	**,533**	**,188**	**,227**	**–,434**	**–,600**	**1,000**

Sig. (1-tailed)	MELA	.	**,130**	**,001**	**,014**	**,123**	**,000**

	WM	**,130**	.	**,264**	**,366**	**,220**	**,136**

	VOC	**,001**	**,264**	.	**,080**	**,474**	**,092**

	MONO	**,014**	**,366**	**,080**	.	**,002**	**,004**

	BI	**,123**	**,220**	**,474**	**,002**	.	**,000**

	MULTI	**,000**	**,136**	**,092**	**,004**	**,000**	.


**Table 10:** Coefficients^a^ table of the multiple regression for gifted participants.


MODEL		UNSTANDARDIZED COEFFICIENTS		STANDARDIZED COEFFICIENTS	T	SIG.	CORRELATIONS		
	
		B	STD. ERROR	BETA			ZERO-ORDER	PARTIAL	PART

1	(Constant)	**81,748**	**6,927**		**11,801**	**,000**			

	VOC	**,878**	**,284**	**,412**	**3,090**	**,004**	**,511**	**,474**	**,401**

	MULTI	**13,758**	**4,170**	**,439**	**3,299**	**,002**	**,533**	**,498**	**,428**


a. Dependent Variable: MeLA.

In the second model of the study for gifted participants, the variables of vocabulary size and multilingualism were observed as significant predictors.

## Discussion

**Figure 5:**
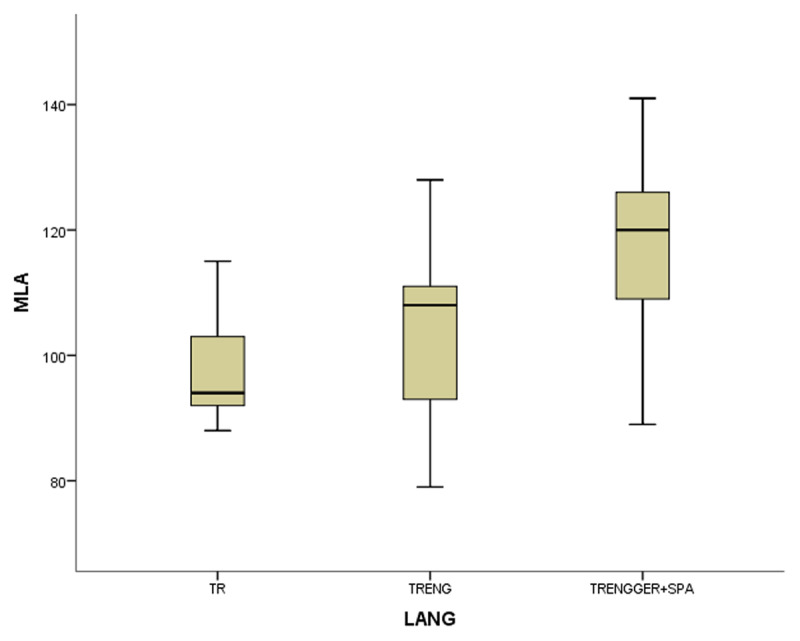
The boxplot of MeLA of gifted participants.

**Figure 6:**
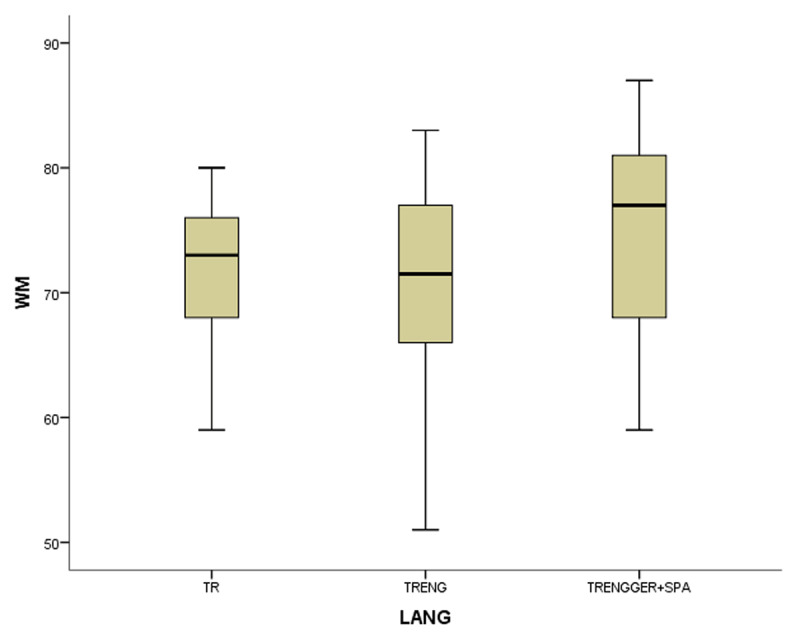
The boxplot of WM of gifted participants (TR for monolinguals, TRENG for bilinguals and TRENGGER+SPA for multilinguals).

**Figure 7:**
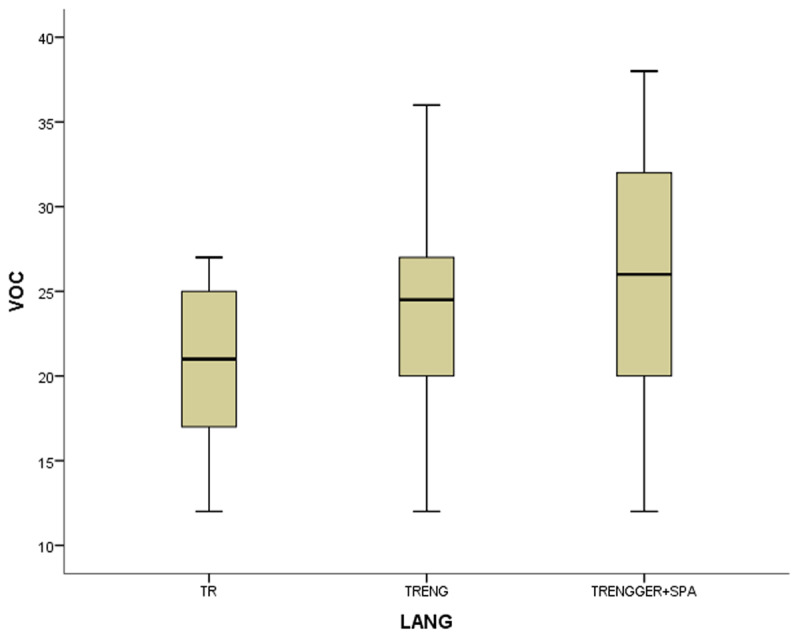
The boxplot of VOC-vocabulary variable of gifted participants.

**Figure 8:**
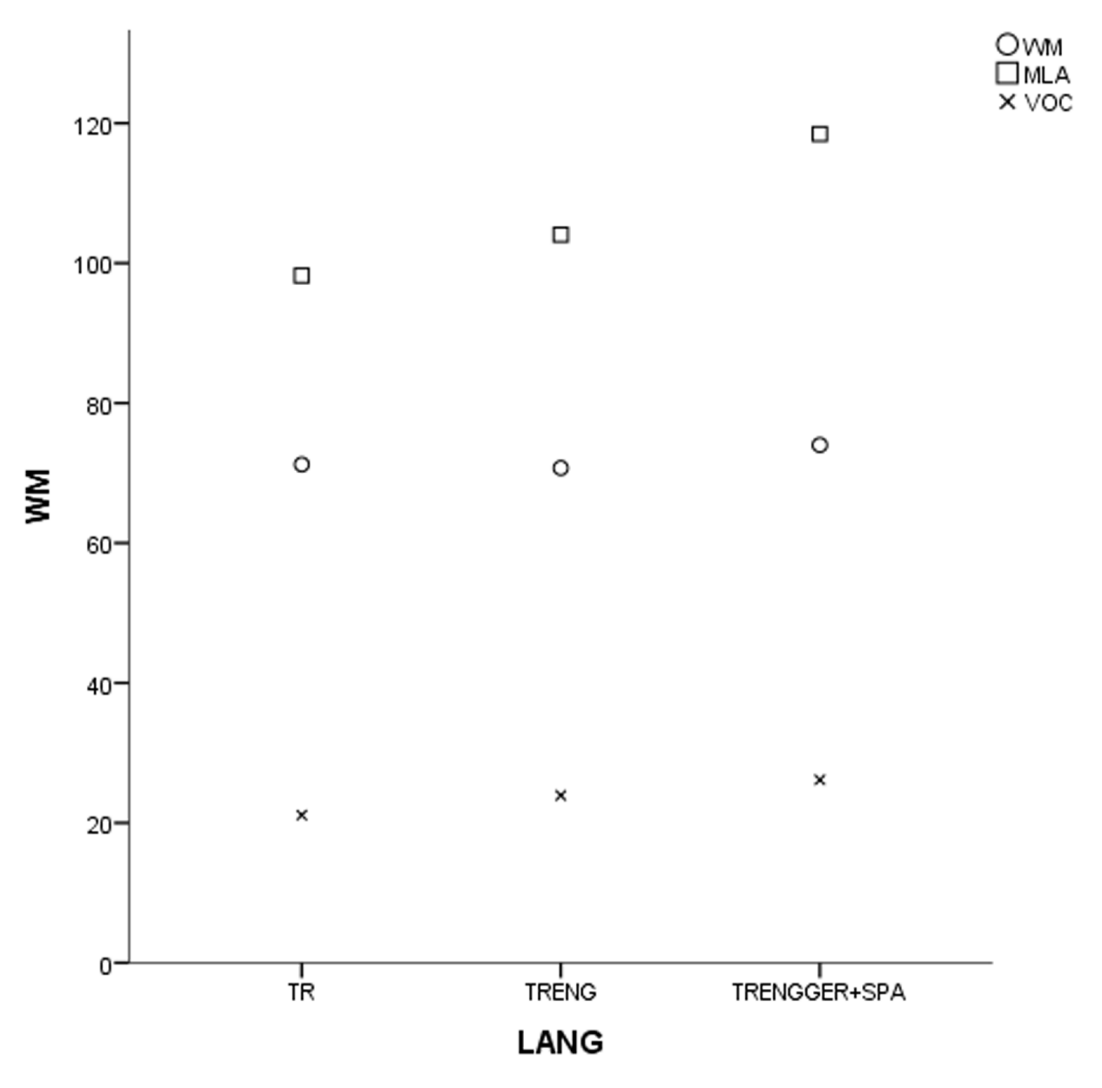
Grouped scatter-plot of the variables in gifted participants (TR for monolinguals, TRENG for bilinguals and TRENGGER+SPA for multilinguals).

In the first study group of non-gifted participants an increase in MeLA, WM and L1 lexicon size scores was observed parallel to the number of languages learnt as indicated in ***Figures 1, 2, 3*** and ***4***. Additionally, multilingual participants in both groups could outperform their bi- and monolingual peers in all three tests. Unlike MeLA and lexicon size, WM was not observed as a significant predictor in the gifted group. This result can be interpreted in two ways; first, Ackerman, Beier and Boyle (2005) argued that the constructs of working memory and general intelligence were not isomorphic. For a second reason, when analysis was conducted on MeLA and multilingualism on the observed level, WM may not explain more variance in the model. Furthermore, positive correlations were observed between multilingualism and MeLA, WM and L1 lexicon size test scores. In order to clarify whether multilingual learning or high IQ level in gifted participants had any positive impact on the scores and which one was a better predictor in high MeLA, (***Figures 5, 6, 7*** and ***8***) WM and L1 lexicon size, it can be stated that multilingual participants in both gifted and non-gifted groups outperformed their peers and achieved the highest scores in all tests. It can be concluded that compared to IQ giftedness multilingual learning had positive impact on higher test scores of the participants in the current study.

## Conclusion and Further Studies

The findings of the present study corroborate previous studies such as Thampson (2013) and Rogers et al. (2017) who examined the relationship between multilingualism and foreign language (FL) aptitude and found positive correlations. Planchon and Ellis (2014) also found that bilinguals and learners with previous formal training outperformed monolinguals in FL test (DLAB, Peterson & Al Haik 1976) which can be attributed their higher metalinguistic awareness. It can be stated that one step further than bilingualism advantage, multilingual learning can result in a number of linguistic and cognitive distinctions due to the complex and interrelated structure of two or more linguistic systems in mind. As language learning abilities are enhanced by bi/multilingual experience (Biedro & Campos 2021), it can be proposed that multilingual individuals can have better cognitive management through WM, MeLA and expanded L1 lexicon size. That is, being a bilingual and multilingual generally help people realize and expand their creative potential into ability to communicate in various occasions. That (linguistic) giftedness is a potential to be developed and during this process multilingualism can be an asset in better cognitive and metalinguistic skills can be stated as well. Multilingual learning can be a better predictor in high scores in mentioned tests due to the fact that two or more systems in one mind can accelerate the development of MeLA and WM and can result an enhanced L1 lexicon size.

### A DMM Approach to Linguistic Giftedness

Taking the context of the current study, it can be stated that despite similar interests, however, the fields of gifted education and cognitive development have had little communication (Steiner & Carr 2003). Although a small number of definitions of LG exist, there has not been a model specifically developed to consider the dimensions of LG and the boundary between IQ giftedness. In this frame, it can be stated that the DMM by Herdina and Jessner (2002) is the only model developed specifically to consider how a linguistic system changes in the presence of three or more languages. In the DMM, the concept of multilingual proficiency is defined as a cumulative measure of psycholinguistic systems in contact (LS_1_, LS_2_, LS_3_, etc.) and their interaction as expressed in cross-linguistic interaction (CLIN) and the influence that the development of a multilingual system shows on the learner and the learning process. Thus, the learner develops skills and qualities that cannot be found in an inexperienced learner and this change of quality in language learning is seen in connection with the catalytic effects of third language learning. Within this construct of multilingual proficiency, heightened level of MeLA is defined as a part of the M(ultilingualism)-factor which includes cognitive factors such as an enhanced monitor and the catalytic effect of third language learning which can be expected to become apparent with growing language learning experience (Jessner 2006). In Dolas’ (2021) Dynamic Model of Linguistic Giftedness (DMLG) which is principally based on the DMM (Herdina & Jessner 2002), the focus is on developmental processes of converting the linguistic potential into a specific and dynamic metalinguistic ability and raising MeLA for the languages in contact in mind. In the centre of the model, MeLA is regarded as an indicator of linguistic giftedness which can be detected through MeLA test scores without assuming a high-level IQ.

Consequently, Jessner (2018) states only if we move away from a simplistic picture of language learning by taking the hyper complexity of the multilingual mind into consideration will we be able to make progress in understanding how language learning takes place. In this frame, the current study is the first study focusing on the relation between multilingualism and linguistic giftedness and is expected to shed light on the complexity and the interconnectedness of the processing mechanisms that characterize learners of multiple languages. The correlation between cognitive and linguistic components of a multilingual mind in gifted language learners’ context is expected to direct the future studies to focus on cognitive opportunities of multilingualism. The current study also indicated the need for an updated explanation of linguistic giftedness and language aptitude by suggesting a new model, the DMLG (Dolas 2021), which can provide a new dimension in multilingualism and cognition studies in a dynamic perspective.

## Limitations

In Turkish language education context, multilingual education has not been supported in state schools due to various economic and educational issues. On the other hand, as a result of adaptation and adjustment process to the European Union, private schools are allowed to follow multilingual education curricula. Thus, one challenging limitation for the current study was that the number of the mentioned private schools is quite rare which caused a limited number of multilingual participants. In the gifted context the situation is demanding as well. Biedron (2016) states that researching exceptional talents is difficult because such talents are quite rare and it is difficult to assemble a group that would be large enough for statistical analysis. Gifted students are selected according to their IQ level higher than 130. In Turkey, Gifted and Talented Centres have been established by Ministry of Education and the number of the centres per city is appointed according to the population. There was only one Gifted and Talented centre in the context of the current study. One challenging limitation was that legal research permissions on gifted children are limited due to a number of strict procedures by the Ministry of Education. Moreover, the lack of sufficient studies in linguistic giftedness and gifted language learners context cause to reach hardly any academic source stating the relation between cognitive and linguistic components in multilingual contexts.

## Data Accessibility Statements

Raw data is available at *https://bibsearch.uibk.ac.at*
